# Severe IgG4-Related Disease in a Young Child: A Diagnosis Challenge

**DOI:** 10.1155/2015/140753

**Published:** 2015-01-29

**Authors:** Susana Corujeira, Catarina Ferraz, Teresa Nunes, Elsa Fonseca, Luísa Guedes Vaz

**Affiliations:** ^1^Pediatric Department, Centro Hospitalar São João, Alameda Professor Hernâni Monteiro, 4200-319 Porto, Portugal; ^2^Pediatric Pulmonology Unit, Pediatric Department, Centro Hospitalar São João, Alameda Professor Hernâni Monteiro, 4200-319 Porto, Portugal; ^3^Pathology Department, Centro Hospitalar São João, Alameda Professor Hernâni Monteiro, 4200-319 Porto, Porto, Portugal

## Abstract

Immunoglobulin G4-related disease (IgG4-RD) is an increasingly recognized syndrome that can appear with multiple organ involvement, typically with tumor-like swelling, lymphoplasmacytic infiltrate rich in IgG4-positive plasma cells, and elevated serum IgG4 concentrations. We report the case of a 22-month-old female child with failure to thrive and recurrent respiratory tract infections since 8 months of age. Physical examination was normal except for pulmonary auscultation with bilateral crackles and wheezes. Laboratory tests revealed elevated erythrocyte sedimentation rate, and elevated serum IgG and IgG4 with polyclonal hypergammaglobulinemia. Thoracic CT and MRI showed multiple mediastinal lymphadenopathies and a nodular posterior mediastinal mass in right paratracheal location with bronchial compression. Initial fine needle aspiration biopsy was compatible with reactive lymphadenopathy but after clinical worsening a thoracoscopic partial resection of the mass was performed and tissue biopsy revealed lymphoplasmacytic infiltrate and increased number of IgG4-positive plasma cells and a ratio of IgG4/IgG positive cells above 40%. Glucocorticoids therapy was started with symptomatic improvement, reduction in the size of the mass, and decrease of serum IgG4 levels after 6 weeks. There are very few reports of IgG4-RD in children. Long-term follow-up is necessary to monitor relapses and additional organ involvement.

## 1. Introduction

Immunoglobulin G4-related disease (IgG4-RD) is an increasingly recognized syndrome of unknown aetiology comprised of a collection of disorders that share specific pathologic, serologic, and clinical features. It is characterized by tumefactive lesions, a dense lymphoplasmacytic infiltrate rich in IgG4-positive plasma cells, storiform fibrosis, and elevated serum IgG4 concentrations [1–3]. The disease was initially recognized in the pancreas but has now been described in virtually every organ system: the biliary tree, salivary glands, periorbital tissues, kidney, lungs, lymph nodes, meninges, aorta, breast, prostate, thyroid, pericardium, and skin. The histopathological features bear striking similarities across the involved organs [1–9]. However, some organs, like the lymph nodes, show a distinct involvement and histopathological features [7, 10].

The clinical picture is highly heterogeneous and the symptoms are related to involvement of the specific target organ, often in the form of a mass lesion. The epidemiology and prevalence of the disease are poorly described and most epidemiologic studies focus on autoimmune pancreatitis. The majority of patients are men older than 50 years [1, 3, 5].

We report a case of a child with pulmonary manifestations of IgG4-RD.

## 2. Case Presentation

A 22-month-old girl was referred to our pulmonology clinic for recurrent respiratory tract infections. She was born after a 38-week gestation and anthropometric measures at birth were normal. She had history of gross motor development delay having sat at 10 months of age and walked independently at 20 months of age.

Recurrent respiratory tract infections started at 7 months of age and were associated with failure to thrive. These respiratory infections were frequently associated with fever and wheezing and treated with inhaled short-acting beta 2 agonists. Despite treatment with montelukast and weekly respiratory physiotherapy, she persisted with chronic morning cough.

On physical examination, there were bilateral crackles and wheezes on pulmonary auscultation and her weight and height were at the 10th percentile. There were no palpable lymph nodes and no abdominal masses.

Complete blood count revealed normal haemoglobin (12.2 g/dL), normal platelet count (256 × 10^9^/L), and peripheral eosinophilia (0.72 × 10^9^/L) and there was polyclonal hypergammaglobulinemia. Blood biochemistry with liver and renal function, iron metabolism, lactate dehydrogenase, and thyroid function tests were normal (serum total protein 80.4 g/L, AST 36 U/L, ALT 15 U/L, alkaline phosphatase 272 U/L, LDH 250 U/L, urea 20 mg/dL, creatinine 0.20 mg/dL, sodium 136 mEq/L, potassium 4.2 mEq/L, chlorides 104 meq/L, calcium 5.1 mEq/L, TSH 2.01 *μ*UI/mL, and T4 1.17 ng/dL). C-reactive protein was normal (2.9 mg/L) and erythrocyte sedimentation rate was elevated (32 mm/hr). The serum angiotensin converting enzyme was normal (22 U/L).

Immunological testing presented with elevated serum immunoglobulin (Ig) G (1690 mg/dL), elevated IgG4 (805 mg/dL) and IgE (127 kU/L), normal IgA (61 mg/dL), normal IgM (107 mg/dL), normal complement levels, and normal neutrophil oxidative burst tests.

**Figure 1:**
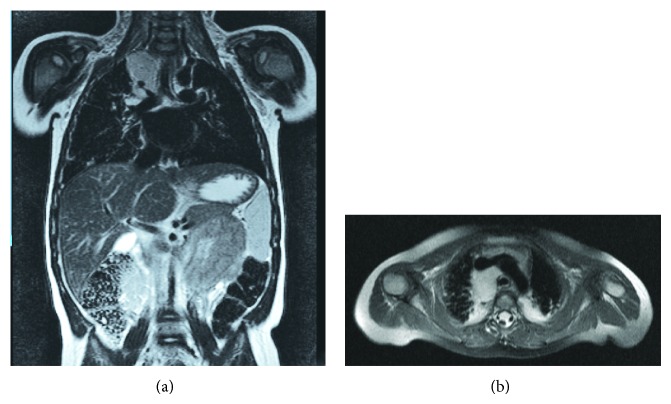
(a) Coronal T2 MRI: complex nodular posterior mediastinal mass infiltrating the right hilum; (b) axial T2 MRI: bilateral mediastinal and hilar lymphadenopathies.

Thoracic CT scan showed multiple right mediastinal and hilar lymphadenopathies, a nodular posterior mediastinal mass (21 × 15 mm) in the right paratracheal region, and bronchovascular consolidation of the right lung base but also of the right upper and medial lobe. Thoracic MRI confirmed the presence of a complex nodular posterior mediastinal mass (34 × 22 mm) infiltrating the right hilum along with consolidation of areas in both of the upper lobes and the right lower lobe suggestive of atelectasis (Figure 1). Bronchofibroscopy examination revealed abundant purulent secretions in the right bronchial tree without visualization of bronchial compression. Bronchoalveolar lavage was negative for infectious aetiologies and cytological examination excluded malignancy and the presence of Cd1a positive cells. Fine needle aspiration biopsy was compatible with reactive lymphadenopathy.

**Figure 2:**
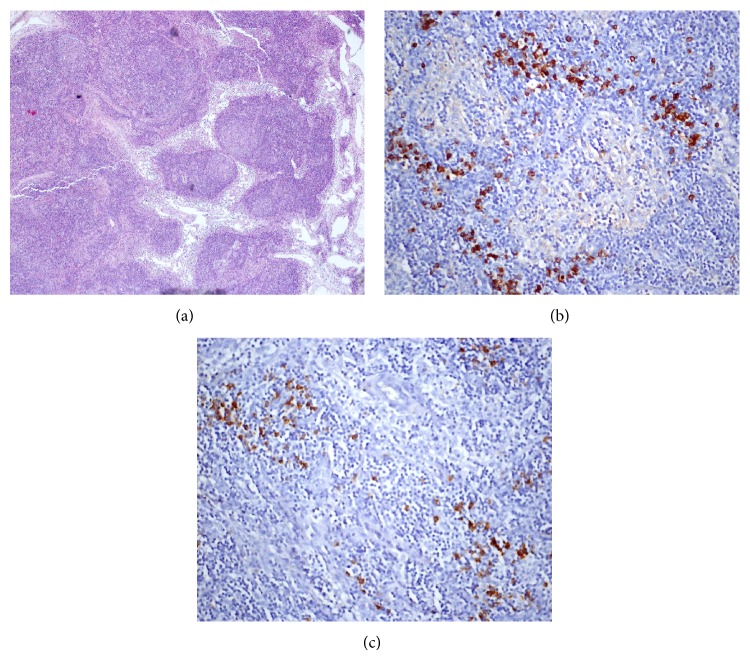
Mediastinal mass biopsy showed reactive lymph nodes presenting follicular hyperplasia with germinal centers and plasmacytic infiltrate ((a) H&E stain, 40x; (b) immunohistochemical stain for IgG, 400x; (c) immunohistochemical stain for IgG4, 400x).

One month later, she continued to have chronic cough and wheezing. A thoracoscopic partial resection was performed of a conglomerate of lymph nodes (30 × 25 × 10 mm). Tissue biopsy histopathological examination revealed follicular hyperplasia with germinal centers. A plasmacytic infiltrate was present in some areas. The number of IgG4-positive plasma cells was high, with 16 IgG4-positive plasma cells/high power field (HPF) and a ratio of IgG4/IgG positive cells over 40% (Figure 2). Histopathological examination of the biopsy sample excluded signs of malignancy and sarcoidosis due to the absence of noncaseating granulomas.

Immunodeficiency, infectious diseases, tuberculosis, cystic fibrosis, malignancy, lymphoma, sarcoidosis, and Castleman's disease were excluded.

After resection there was worsening of respiratory symptoms and two episodes of pulmonary infection treated with systemic antibiotic therapy. Thoracic MRI, repeated 4 months after resection, showed increase in the size of the right paratracheal mediastinal mass compared to its initial dimensions (30 × 19 mm) and was persistence of subsegmental bilateral atelectasis.

Glucocorticoid therapy was started (prednisone 2 mg per kilogram) with clinical improvement and decrease of serum IgG4 levels (226 mg/dL) after 6 weeks. Significant reduction of the mass size was confirmed by MRI which showed a small right tracheal nodular image (7 mm). Glucocorticoids were tapered over a period of six months. The patient has been clinically stable for 12 months after stopping therapy, with height and weight at the 50th percentile at 4 years old.

## 3. Discussion

Pulmonary involvement in IgG4-RD has been reported with a broad spectrum of intrathoracic findings. These manifestations appear to be rather heterogeneous resulting from involvement of the lung parenchyma, intrathoracic lymph nodes, mediastinum, and pleura [4, 9]. Lung parenchymal involvement consists mainly of rounded opacities and interstitial lung disease. Airway disease can be caused by extrinsic compression of the central airways due to fibrosing mediastinitis and bronchiectasis. Pleural disease consisted of nodular lesions in the visceral or parietal pleura, pleural effusion, and pleuritis [5, 9]. The most common intrathoracic manifestation is mediastinal and hilar lymphadenopathy, which has been described in 40% to 90% of patients with IgG4-RD. Intrathoracic manifestations have been reported in the presence or absence of one or more extrapulmonary lesions, such as autoimmune pancreatitis [5, 8, 9, 11].

Lymphadenopathy can develop subsequent to the diagnosis of extranodal IgG4-RD or it can be the initial presentation of the disease [3–5]. When lymphadenopathy is generalized, constitutional symptoms are usually absent and lactate dehydrogenase level is normal. Differential diagnosis is broad and includes lymphoma, sarcoidosis, Castleman's disease, or disseminated malignancy [3, 5, 6].

In other patients with IgG4 –RD lymphadenopathy increased serum levels of IgG, IgG4, and IgE, polyclonal hypergammaglobulinemia, elevated sedimentation rate, and positive autoantibodies have also been reported [3, 7, 10]. Many patients have allergic features such as atopy, eczema, asthma, and modest peripheral blood eosinophilia [1, 3].

The majority of patients with IgG4-RD have elevated serum IgG4 concentration (>135 mg/dL). However, elevated IgG4 may also be observed in other diseases suggesting that high serum IgG4 is not a specific marker of IgG4-RD [1, 5, 7].

The diagnosis of IgG4-RD requires both characteristic histopathological features and increased number of IgG4 positive plasma cells or an elevated IgG4 : IgG ratio in tissue. The major histopathological features observed in several organs are a dense lymphoplasmacytic infiltrate, storiform fibrosis, obliterative phlebitis, and eosinophil infiltrate [1, 2, 5, 10]. However, the lymph nodes are an exception to this rule as storiform fibrosis and obliterative phlebitis may be inconspicuous or absent. Therefore, the histopathological diagnosis relies considerably on the number of IgG4 positive cells and on the ratio of IgG4 positive/IgG positive plasma cells [2, 5, 10]. Five histological patterns have been reported in the literature associated with IgG4-related lymphadenopathy: multicentric Castleman disease-like, follicular hyperplasia, interfollicular expansion, progressive transformation of germinal centers, and nodal inflammatory pseudotumor-like. Reactive follicular hyperplasia is a common histopathological finding in lymph nodes biopsies [2, 3, 5, 10, 12].

The appropriate cut-off point of the number of IgG4 positive plasma cells varies in different organs, but the presence of >10 IgG4 positive plasma cells/HPF on biopsy specimens has been proposed as diagnostic feature. However, IgG4 positive plasma cell count alone may not help to distinguish between IgG4-RD and other disorders [1, 2, 5, 7, 9]. The IgG4 positive/IgG positive plasma cell ratio of >40% is a comprehensive cut-off value in any organ and is very suggestive of the diagnosis [2, 5, 7].

The comprehensive clinical diagnostic criteria for IgG4-RD were fulfilled: (1) mediastinal and hilar lymphadenopathy, (2) elevated serum IgG4 concentrations, and (3) histopathologic examination with lymphoplasmacytic infiltration and >10 IgG4 positive plasma cells/HPF and IgG4 positive/IgG positive plasma cell ratio >40% [13].

There are very few reports of IgG4-RD in children and only one in a 15-year-old boy with lung involvement to our knowledge [14]. IgG4-related lymphadenopathy is often asymptomatic and may not require immediate treatment but our patient was very young and had significant lung disease with severe systemic repercussion and failure to thrive.

Benign/reactive lymph nodes are more common in children but are less frequently excised compared to adults. Differential diagnostic is broad and although the findings have some distinctive features, in many circumstances, they may not be sufficiently distinctive as to exclude a diagnosis of IgG4-related lymphadenopathy.

Multiorgan disease may be evident at diagnosis but also can evolve metachronously over months to years. Spontaneous improvement is reported in a minority of cases but not in intrathoracic IgG4-RD [1, 5, 9].

No randomized treatment trials have been conducted, particularly in children. Glucocorticoids are the first line of therapy and most IgG4-RD patients respond favourably to this treatment. Most centers start with prednisolone at a dose of 0.6 mg per kilogram of body weight or 40 mg for 2 to 4 weeks and taper the dose over a period of 3 to 6 months, although some authors suggest to continue at a dose between 2.5 and 5 mg per day for up to 3 years [1, 5, 6, 9, 15].

Our patient had a good clinical response to glucocorticoids but durability of treatment response is unclear after prednisolone tapering and relapse is frequent. A major determinant of treatment responsiveness is the degree of fibrosis, which was absent in this case [1, 5]. Serial measurements of IgG4 concentrations have been proposed as indicator of disease activity but although IgG4 concentrations become lower with glucocorticoid treatment, they remain above normal value in most patients [1, 3, 5].

The natural history of IgG4-RD has not been well defined, particularly in paediatric patients. Long-term follow-up is necessary to closely monitor relapses and additional organ involvement. Other courses of steroid therapy may be needed and eventually immunosuppressive therapy.
